# A Virtual Reality–Based mHealth App to Enhance Health Care Skills and Promote Health Equity by Empowering Health Care Professionals to Provide Empathetic, Compassionate, and Unbiased Patient Care Through Digital Experiential Learning: Qualitative Study

**DOI:** 10.2196/64147

**Published:** 2025-10-10

**Authors:** Dixit Bharatkumar Patel, Mayank Bharatkumar Patel, Thomas Wischgoll, Yong Pei, Paul J Hershberger

**Affiliations:** 1 Department of Computer Science & Engineering Wright State University Dayton, OH United States; 2 Fabricators Incorporated Chattanooga, TN United States; 3 Department of Computer Science Kennesaw State University Marietta, GA United States; 4 Department of Family Medicine Wright State University Dayton, OH United States

**Keywords:** mHealth app, experiential learning, health care skills, health care, mobile health, serious games, health equity, virtual reality, role-playing games, health care professionals

## Abstract

**Background:**

Health care professionals’ educational preparation and practices significantly influence care experiences and health outcomes. Deficient awareness of the impact of stereotypes, biases, prejudices, and social determinants of health (SDH) can lead to negative care experiences, strained health care professional-patient relationships, and health disparities. Addressing these challenges necessitates enhancing health care professionals’ skills, including inclusive communication, cultural humility, recognition of SDH, and fostering empathy and compassion, promoting health equity and better care experiences.

**Objective:**

This research aims to introduce a mobile health (mHealth) app designed using a digital experiential learning (DEL) approach to strengthen health care professionals’ competencies, thereby improving patient care experiences and promoting health equity. The key objectives are to deliver essential health care skills, such as cultivating cultural humility, developing inclusive communication proficiencies, understanding the lasting impact of SDH, comprehending how implicit and explicit biases affect health outcomes, fostering compassionate and empathetic clinical attitudes, and promoting continuous professional development. Together, these aims advance patient-centered care and help reduce health disparities.

**Methods:**

The mHealth app integrates virtual reality–based serious role-playing hypothetical scenarios and a life course module to provide health care professionals with immersive first-person learning experiences. The scenarios include a Syrian refugee with limited English proficiency and an African American pregnant woman with a history of opioid use disorder, each lasting ≈30 minutes to deliver essential health care skills. The pre- and postassessment questionnaires are integrated within the app to measure the learning outcomes of diverse professionals who voluntarily engaged with the app. Distinct hypotheses were formulated and evaluated, referring to specific questionnaire items to assess the app’s impact on professionals’ skills and attitudes.

**Results:**

The mHealth app significantly enhanced health care professionals’ skills and attitudes, including increased confidence, preparedness for patient interactions, awareness of the lasting impact of SDH, positive beliefs, reduced prejudice, improved perspectives on patient responsibility and external factors, as well as greater compassion and empathy. These outcomes, obtained through evaluating distinct hypotheses and analysis supported by multiple statistical approaches, including CI analysis, Cohen *d* effect sizes, odds ratios, 1-tailed paired *t* tests, and descriptive response distributions, directly align with the study’s objectives, fostering health equity and patient-centered care. Overall, the app effectively improved health care professionals’ competencies, contributing to better care experiences and outcomes while promoting health equity.

**Conclusions:**

This study delivers a comprehensive data-driven evaluation that validates the effectiveness of the mHealth app in enhancing health care professionals’ skills and fostering health equity. It addresses challenges in health care education by delivering a scalable, accessible, and immersive learning platform. Supported by virtual reality technology and an experiential learning framework, the mHealth app presents a promising avenue to empower health care professionals with skills to provide patient-centered care in diverse, complex settings.

## Introduction

### Background of Study

The Centers for Medicare & Medicaid Services NHE (National Health Expenditure) Fact Sheet highlights the growing demand for health care [1], emphasizing the need for equitable access. It is essential that individuals receive appropriate care without the influence of implicit or explicit biases stemming from sociodemographic factors such as race, ethnicity, sex, age, poverty, disability status, or geographic location [2].

Research shows that health care professionals are vulnerable to biases, which contribute significantly to health disparities and require urgent attention [3]. These biases, particularly toward those outside one’s social circle, tend to form over time and are frequently negative in nature [4]. If left unaddressed, such biases can result in inequitable treatment of marginalized populations [5,6]. For example, Hoffman et al [7] found that African Americans may receive less pain medication compared with White Americans, highlighting how bias affects clinical decisions.

In addition to genetics and lifestyle choices, social determinants of health (SDH)—defined as the conditions in which people are born, grow, work, live, age, and engage in recreation—have a significant impact, accounting for 30% to 55% of health outcomes [8,9]. Health care providers who acknowledge their biases and apply skills such as perspective-taking and individuation can help reduce disparities [10-14]. Therefore, it is crucial to raise awareness among physicians, physician assistants, social workers, nurses, pharmacists, and other health care professionals about essential patient-centered skills. These include cultural humility, inclusive communication, understanding the impact of SDH, recognizing how bias affects health outcomes, and promoting empathy. Such competencies improve patient experiences, minimize biased health care encounters, and advance health equity.

### Literature Review

In health care education, developing intellectual capacity and behavioral awareness is essential for fostering equitable and patient-centered care. Education plays a vital role not only in enhancing logical reasoning but also in shaping individuals’ beliefs and social interactions, which are closely tied to how they perceive and respond to diverse patient populations. Hands-on learning experiences play a significant role in elevating an individual’s competency level [15].

One foundational approach to hands-on education is experiential learning theory, introduced in 1984 by educational theorist Kolb [16]. Experiential learning theory integrates the learning styles inventory and the experiential learning cycle to emphasize that knowledge is created through the transformation of experience. Kolb’s work [16], influenced by researchers such as John Dewey, Kurt Lewin, and Jean Piaget, highlights that learning involves grasping and transforming experiences, a concept especially relevant in training health care professionals to engage in self-awareness, perspective-taking, and bias reduction.

**Figure 1:**
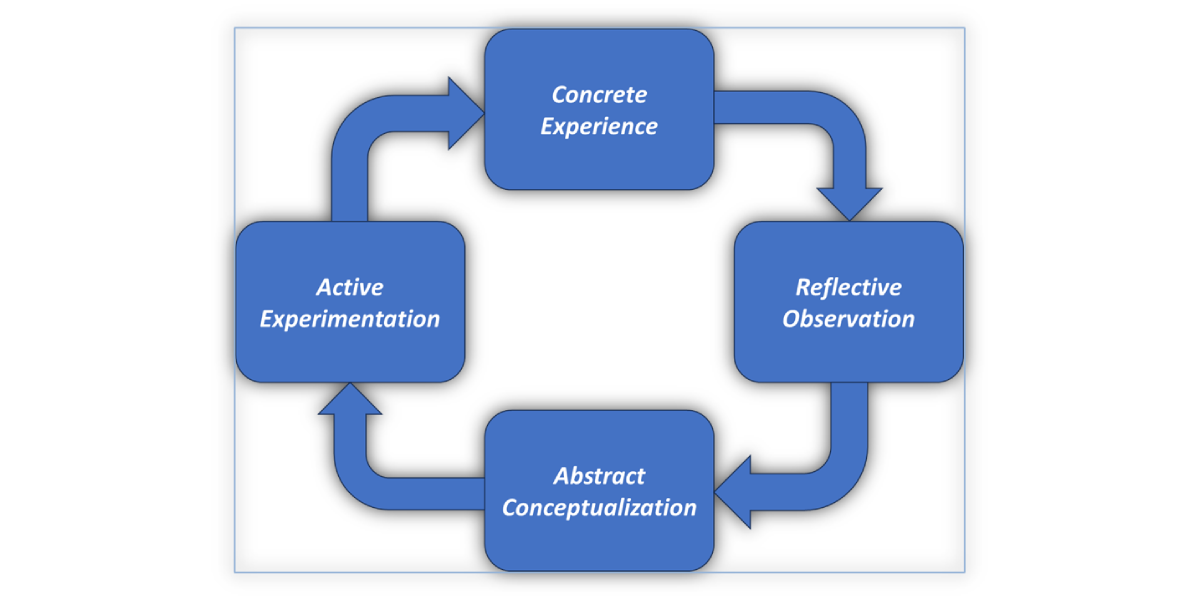
Illustration of Kolb’s experiential learning cycle [16], depicting the 4 stages of learning—Concrete Experience, Reflective Observation, Abstract Conceptualization, and Active Experimentation—central to the design of immersive health care education experiences.

As illustrated in Figure 1, the experiential learning cycle, learners begin by engaging in direct experiences, reflect upon them, form abstract generalizations, and apply these insights through experimentation—fostering deeper understanding and skill acquisition [16]. Integrating this model into digital platforms empowers learners to apply knowledge in simulated environments and clinical scenarios.

Hence, in this study, the “Digital Experiential Learning” (hereinafter referred to as DEL) term refers to an approach that harnesses digital technologies to create immersive and hands-on learning experiences. It integrates the principles of experiential learning [16], where learners actively engage with real-world tasks and reflect on their experiences, with digital tools and platforms to enrich the learning process. This approach uses various digital mediums such as simulations, virtual environments, interactive multimedia, and web-based collaborative platforms to facilitate exploration of complex concepts, development of practical skills, and acquisition of meaningful insights. The efficacy of DEL has been demonstrated in fields such as tourism education, where virtual field trips and simulations significantly enhanced learners’ socioemotional engagement and content retention [17], and in sustainable horticulture and landscape management, where augmented and virtual reality (VR) technologies promoted team-based learning and environmental awareness within Kolb’s experiential learning framework [18]. While DEL promotes active participation, problem-solving, and critical thinking, it also presents challenges such as access to technology and the need for digital literacy. Nonetheless, its dynamic and interactive nature enhances understanding and retention of knowledge across diverse educational settings.

Within this digital landscape, serious games have emerged as powerful educational tools. Serious games are digital games in which hidden or serious objectives are seamlessly integrated into the digital app [19]. These games embed learning objectives into engaging formats, providing motivational and learning experiences [19]. Thus, serious games are a powerful concept that can be used to deliver motivational and empathetic learning experiences for individuals within specific fields, including health care. Enhancing this notion further, role-playing games (RPGs) allow learners to embody characters in simulated settings, deepening empathy, intuition, and contemplation—particularly in clinical training [20,21]. Digital RPGs have shown success in teaching educational and social skills [22,23], and their adoption in occupational universities has improved standards in technology-enhanced learning [24]. Building on these concepts, Patel et al [25-27] have demonstrated the value of computer-supported expert-guided experiential and cognitive learning tools or mobile health (mHealth) apps for training health care professionals. These tools help cultivate nonverbal communication and behavior-modification skills crucial for interactions with individuals on the autism spectrum and in general health care contexts [25-27].

The evolution of technologies has accelerated, driving a shift from traditional learning to technology-based learning in higher education, including health care education [28]. Studies reveal that perceived usefulness, ease of use, and health benefits influence user adoption of mHealth apps [29]. Innovations like the integration of wearable devices and smartphones enable real-time health management and monitoring [30].

Several reviews have evaluated mHealth apps in health care delivery. Ameyaw et al [31] highlighted their effectiveness in maternal health—addressing anxiety, diabetes, weight management, and behavioral risks—though most implementations occurred in high-income regions, underscoring a need for global equity. Iribarren et al [32] found modest benefits of mHealth over standard care but emphasized the variability in app impact and the importance of rigorous design and evaluation methods. Lee et al [33] reviewed physicians’ use of mobile apps for communication, education, monitoring, and clinical decision-making, identifying both advantages and challenges related to privacy and distraction. Similarly, Liu et al [34] found that hypertension-focused mHealth tools, while patient-centered, also supported provider education and chronic disease management. The PsyCovidApp, studied by Fiol-DeRoque et al [35], demonstrated effectiveness in reducing health care workers’ stress during the COVID-19 pandemic—benefiting performance and well-being. Jong et al [36] explored nurses’ smartphone use, finding improvements in access to medical information, alongside concerns about distraction and data privacy.

Additionally, a concept of “educational game design based on experiential learning theory” was introduced by Li et al [37], suggesting that incorporating experiential learning elements into educational games can enhance students’ motivation and interest in learning. However, while experiential learning—often summarized as “learning by doing”—is a proven educational approach [16], direct implementation in high-risk, real-world health care settings may not be appropriate or safe [38]. Inadequate learning experiences and insufficient training can lead to suboptimal care and weaken provider-patient relationships. Therefore, developing structured digital tools that facilitate DEL offers a safer, immersive, and more effective educational experience—empowering health care professionals to build critical competencies before applying them in clinical practice.

### Study Objectives and an Innovative mHealth App Solution

In today’s evolving health care landscape, equipping professionals with essential skills—such as inclusive communication, cultural humility, empathy, and an understanding of enduring impacts of SDH, biases, and prejudices on health outcomes—are critical to improving care quality. Providing coaching tools and expert-guided experience enhances professional competence and supports equitable, patient-centered health care delivery. Innovative approaches to experiential learning help make these resources more accessible—regardless of location, schedule, or professional role—thus democratizing health care education and empowering both providers and communities.

To address these challenges, this study integrates experiential learning into digital platforms—leveraging VR and serious RPG features—to support the development of health care skills. The objectives include the following. (1) Facilitating the delivery or enhancement of essential health care skills. (2) Addressing the challenge of distributing effective and efficient educational mediums in the health care sector. (3) Maximizing learning outcomes by providing a first-person experiential learning approach. (5) Developing a scalable product that can be accessed and used on mobile devices at any time and from anywhere, with only an internet connection and no additional costs. (6) Reducing the reliance on real-life standardized patients and expert trainers for educational and clinical practices. (7) Promoting continuous professional development for health care professionals. (8) Enhancing patient-centered care by fostering confidence and preparedness for real-life patient interactions, positive beliefs, compassion, empathy, and nonprejudiced attitude toward a patient or an individual. (9) Improving patients’ health outcomes and overall health care quality through immersive and interactive learning experiences. (10) Increasing awareness and understanding of the impact of SDH on patient care. (11) Addressing implicit biases and promoting cultural humility in health care delivery. (12) Creating a safe and controlled learning environment for practicing high-risk care scenarios.

In addition, the app strengthens core clinical competencies through realistic case simulations. Specifically, the educational content supports the development of the following learning outcomes:

- Deepened understanding of patients’ lived experiences
- Recognition of how SDH intensifies patient challenges
- Awareness of implicit biases and their influence on clinical interactions
- Empathy-driven care, fostered through reflection on unique patient narratives

To achieve these objectives, an mHealth app [39,40] was developed based on DEL concepts (hereafter referred to as “the app” or “the mHealth app”). The app functions as a serious RPG and features 2 virtual case scenarios: one involving a Syrian refugee with limited English proficiency, and another centered on an African American pregnant woman with a history of opioid use disorder. These immersive simulations allow learners to practice clinical decision-making and empathetic interaction in a safe and controlled environment. The app also integrates an interactive life course module, adapted from content originally developed by CityMatCH and later adapted by Wright State University [41]. This feature guides users through the life journey of virtual patients, highlighting the long-term effects of SDH on health outcomes and clinical encounters. Embedded within the app are 2 assessment surveys designed to capture user feedback and evaluate learning outcomes—allowing for continuous improvement of the educational platform.

In contrast to earlier pilot efforts, this study includes a robust sample size of 240 participants and presents a detailed analysis of the app’s design, backend workflow, and educational impact. Comprehensive survey data and hypothesis testing offer rich insights into user engagement and knowledge acquisition. Ultimately, this DEL-based mHealth app provides a scalable and innovative solution to health care education challenges—advancing both clinical skills and health equity through immersive, technology-enabled learning.

## Methods

### Ethical Considerations

This project was determined to be exempt by the Wright State University (IRB record #06659) and the Ohio State University (10-24-2018) Institutional Review Boards (IRBs). This study involved human participants and received a category 3b exemption from the Institutional Review Board of Wright State University. The study was supported in part by the Ohio Department of Medicaid (grant #60077777) as part of the Medicaid Equity Simulation Project, administered by the Ohio Colleges of Medicine Government Resource Center. Informed consent was obtained within the app prior to participation. Participant privacy and data confidentiality were strictly maintained throughout the study. All procedures were conducted in accordance with institutional guidelines. No compensation was provided to participants.

### Study Approach

This research leverages recent advancements in serious games, VR, human-computer interaction, DEL, and mobile computing to achieve objectives. The study integrates a DEL approach into a VR-based serious RPG or mHealth app. This integration harnesses the multimodal capabilities of cost-effective Android and iOS smart devices, ensuring widespread accessibility and maximizing the delivery of health care skills and awareness.

Incorporating a first-person viewing experience within the DEL medium enhances the learning process by providing health care professionals and individuals with immersive and enduring knowledge acquisition. The use of VR-based hypothetical case scenarios featuring diverse virtual characters enables safe and risk-free learning experiments, eliminating potential harm to real-life patients during clinical practice.

A key strength of this approach is the incorporation of educational objectives within an entertaining gameplay mechanism. This combination facilitates self-driven and self-motivated learning, empowering learners to actively engage in the educational process. As a result, cognitive learning and behavioral change are promoted. Additionally, the inclusion of visual cues within the gameplay reduces the complexity of the learning mechanism, minimizing the need for extensive explanatory materials while ensuring an effective learning experience.

To assess the effectiveness of the DEL approach–based tools in the health care domain, the mHealth app incorporates pre- and postsurvey or assessment questionnaires within the gameplay. This facilitates an analysis of user learning performance and evaluation of the overall effectiveness of the DEL approach in health care education. Furthermore, this research seeks to advance health care education by providing an immersive, engaging, and effective learning platform that enhances health care professionals’ skills and knowledge. The integration of innovative methodologies underscores the potential to transform health care education, delivering a novel and impactful learning experience.

### mHealth App Design Framework and Implementation Strategy

#### Overview

The primary objective of this research is to design and develop a DEL approach-based mHealth app or a serious RPG that empowers and inspires health care professionals to acquire advanced health care experiential learning through the convenience of mobile platforms, specifically iOS and Android devices. By leveraging the capabilities of widely accessible and cost-effective smart devices like iPhones, iPads, Android phones, and tablets, the app maximizes the potential to effectively deliver health care skills and awareness.

The app design centers around 2 hypothetical case scenarios, each featuring a central virtual character or patient. The first case scenario revolves around Muhammad Ayoubi, a Syrian refugee with limited English proficiency (hereinafter referred to as Muhammad’s case), while the second case scenario focuses on Ebony Williams, an African American pregnant woman with a history of opioid use disorder (hereinafter referred to as Ebony’s case). These scenarios provide health care professionals and other learners with an engaging and immersive opportunity to experience a patient’s clinical visit from a first-person perspective. By actively participating in these scenarios, learners can enhance their health care skills and contribute to the objectives of this research.

#### High-Level Block Diagram Illustrating the mHealth App’s Workflow

**Figure 2:**
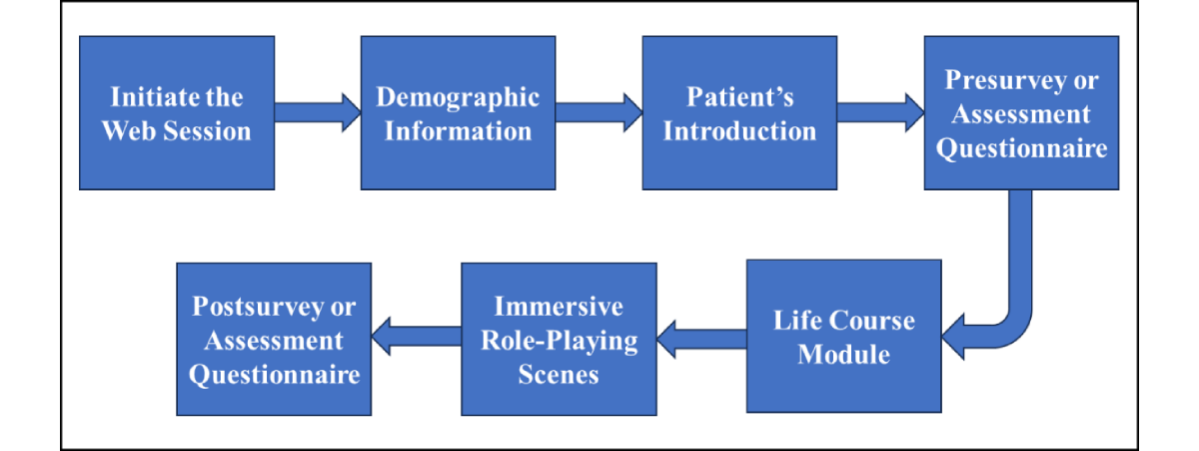
High-level block diagram of the backend architecture and user interaction flow within the mobile health app.

To provide a clear overview of the mHealth app workflow, a high-level block diagram is illustrated in Figure 2. Upon launching the app, a session number (token) is obtained from the web server, which randomly assigns an identifier representing either case scenario (ie, Muhammad’s case or Ebony’s case) to the learner. The session number is essential to begin the app session; without it, the learner cannot proceed in the learning process. This is followed by the collection of demographic data, enabling analysis of participants’ responses in relation to their demographic characteristics. Subsequently, the learner is introduced to the patient, providing a brief overview of their background and reason for the clinical visit based on the assigned case scenario.

During the patient introduction, the learner assumes the perspective of the health care provider, permitting a deeper and more immersive connection with the patient. To gauge the learner’s initial impressions and emotional responses, a presurvey or assessment questionnaire is administered, capturing their thoughts on assuming the role of the health care provider and their perception of the introduced patient.

To further enhance the educational experience, the app incorporates a life course module focused on SDH specific to each virtual patient (Muhammad or Ebony). This module provides valuable insights into the patient’s background, social context, and various factors that contribute to their overall health and well-being.

The app also features immersive VR-based role-playing scenes or sessions, carefully designed to encompass different aspects of health care practice. These scenes include the virtual patient’s preparation for the clinical appointment, transportation to the health center, check-in experience at the facility, and the clinical encounter between the virtual patient and virtual health care provider. Moreover, specific tasks unique to each case scenario are incorporated, such as filling prescriptions in Muhammad’s case and seeking medication-assisted treatment in Ebony’s case. By engaging in these role-playing sessions, health care professionals can acquire advanced experiential learning in a safe and risk-free environment, avoiding any potential harm to real-life patients.

A crucial aspect of the app’s design is the integration of hidden educational objectives within an entertaining gameplay mechanism. This combination ensures that the VR-based serious RPG serves as a self-driven and self-motivational learning tool, enabling learners to undergo cognitive learning and experience behavioral change. The incorporation of visual cues within the gameplay reduces the complexity of the learning process while still delivering an effective and engaging educational experience.

To evaluate the effectiveness of the mHealth app or serious RPG in the health care domain, pre- and postsurvey or assessment questionnaires are embedded within the gameplay. These questionnaires facilitate analysis of learner performance and provide insights into the overall impact of the approach in enhancing health care skills and education.

Thus, this study contributes to the advancement of health care skills and educational awareness by leveraging recent developments in serious games, VR, human-computer interaction, DEL, and mobile computing. The design and development of a VR-based serious RPG provides health care professionals with a transformative learning platform. By immersing learners in realistic virtual scenarios and enabling interaction with diverse virtual characters, the app facilitates effective acquisition and enhancement of essential health care skills.

#### Digital Experiential Learning Approach–Based mHealth App Design and Development Aspects

This section explores the intricate details of the app’s design and development aspects. It outlines the key elements that shape the user experience and functionality, discussing the integration of various modules and features. Through an examination of the technical underpinnings and deliberate design considerations, valuable insights are provided into the creation of a seamless and immersive learning environment.

#### Seamless Integration of Virtual Environment and Virtual Characters

This study harnesses virtual environments and virtual characters to create an engaging and effective learning experience for health care professionals [39,40]. The integration of a virtual environment within the serious RPG served multiple purposes, including providing public education on medical diseases, patient treatment, and training for health care professionals. By immersing learners in real-world scenarios, this approach sustains active engagement with experiential learning modules, enabling them to relate to practical situations while developing essential academic and professional competencies.

**Figure 3:**
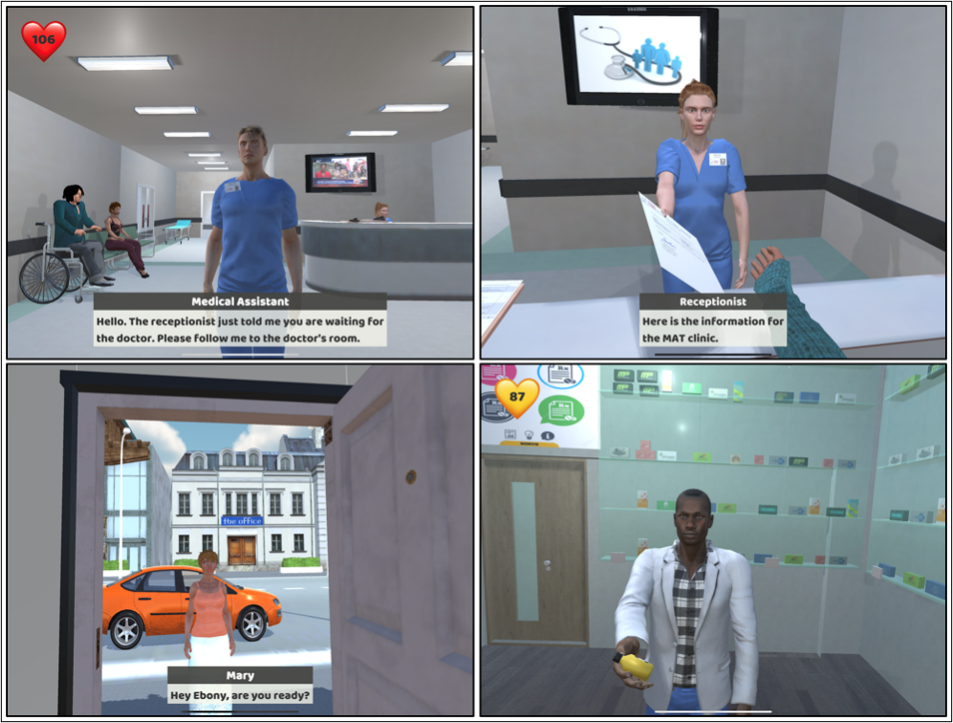
Visual representation of the simulated clinical environment, and animated patient characters featured in the app to facilitate immersive role-playing and empathy-based learning.

Using lifelike virtual objects and intricate real-world representations, learners explored various clinical procedures, enhancing their understanding and proficiency in a risk-free environment. The Unity Game Engine, provided by Unity Technologies, played a pivotal role in developing and integrating the virtual world into the mHealth app, enabling the creation of realistic and immersive role-playing scenarios. Figure 3 shows examples of such scenarios.

In addition to the virtual environment, the integration of virtual characters further enriched the learning experience in the DEL platform. Virtual personas, including patients and peers, provided learners with focused and involved interactions, highlighting expressions and emotions that enhanced gameplay engagement. With well-developed storylines and virtual settings, learners could repeat gaming tutorials for clinical practices, professional communication, and cognitive skill development at their convenience.

Virtual characters played an active role in engaging and conversing with players during the hands-on experience, allowing for meaningful interactions that strengthened learners’ empathy and compassion. Reallusion software tools were used to develop these virtual characters, which were later seamlessly integrated into the Unity Game Engine to create compelling story scenes as illustrated in Figure 3. The combination of the virtual environment and virtual characters elevated the overall learning experience and contributed to the app’s success in empowering health care professionals with practical skills and compassionate attitudes toward patient care.

#### Integration of Interactive VR-Based Serious Role-Playing Sessions

An indispensable aspect of the mHealth app or serious game involves the integration of immersive role-playing scenarios that effectively engage and educate learners in the realm of clinical patient care [39,40]. Through these rigorous role-playing sessions, learners gain insights into patient experiences, the ramifications of SDH, and the implications of biases on health outcomes. Within these interactive sessions, learners assume the role of virtual health care providers and make consequential conversational choices that dictate the virtual patient’s responses, behaviors, and health trajectories. This dynamic conversation flow is facilitated by using N-ary tree data structures and Unity C# scripts, ensuring a fluid and lifelike interaction with the virtual patients, thus delivering an authentic and compelling learning experience.

**Figure 4:**
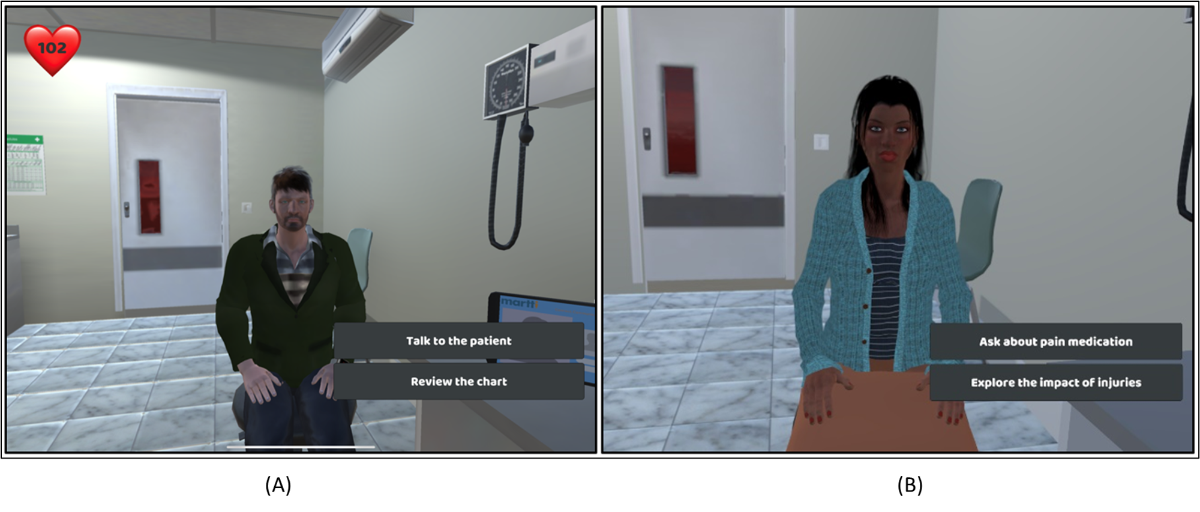
Visual representation of interactive virtual reality–based serious role-playing scenarios embedded within the app. (A) Muhammad’s case: a Syrian refugee with limited English proficiency, and (B) Ebony’s case: an African American pregnant woman with a history of opioid use disorder.

**Figure 5:**
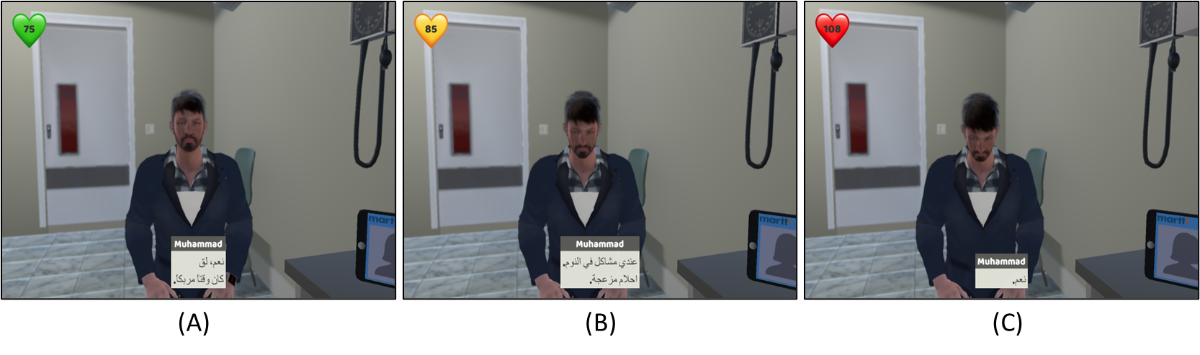
(A) Outcome display when the health care provider selects an “Empathetic” response: virtual patient experiences improved health outcomes, and increased trust and engagement, reinforcing compassionate care approaches; (B) Outcome display from a “Neutral” choice selection by the health care provider, showing moderate patient engagement and incomplete resolution of health challenges—demonstrating the value of effective communication; and (C) Outcome display resulting from an “Apathetic” response, highlighting diminished patient health outcomes and disengagement—used to emphasize the negative effects of provider bias and lack of empathy.

As learners progress through the clinical encounter module, they interact with individual virtual patients, Muhammad and Ebony, each presenting specific challenges and eliciting distinct responses, as highlighted in Figures 4 and 5. The user interface was meticulously designed to offer distinct conversational choices—Empathetic, Neutral, and Apathetic—presented as on-screen buttons. These choices trigger a spectrum of emotions and behaviors in the virtual patient, with their impacts portrayed through an array of cues, including facial expressions, gestures, dialogue captions, and even changes in heart rate. The choices for available responses were designed in a distinct color scheme and shown at a different position to clearly separate them from the captions provided throughout the entire interaction.

A key design decision was the inclusion of the heart rate indicator, an innovative addition that visually represents the patient’s stress level at any given point, as illustrated in Figure 5A. The rationale behind this choice was to provide learners with immediate feedback on the emotional impact of their dialogue choices. For instance, when learners opt for the “Empathetic” choice, the virtual patient maintains direct eye contact, and the green-colored heart rate indicator in the upper left corner, as shown in Figure 5A, reflects a reassuringly normal range, indicating a sense of calm. Conversely, the “Neutral” choice, as demonstrated in Figure 5B, triggers a subtly upset response, with the patient’s bowed head signifying concern. Here, the yellow-colored heart rate indicator, shown in Figure 5B, registers a moderately high range, signaling a heightened emotional state. Finally, the “Apathetic” choice, depicted in Figure 5C, triggers a notably distressed reaction, accompanied by a pronounced head bow. The patient avoids eye contact, and the red-colored heart rate indicator, shown in Figure 5C, signifies a significantly elevated stress level. The meticulous distinction in design between conversational buttons and subtitles ensures clarity in user interaction. The app’s design was guided by expert discussions that underlined the importance of facilitating a nuanced learning experience, reinforcing the significance of empathetic communication and compassionate, unbiased patient care.

Furthermore, the mHealth app encompasses supplementary role-playing sessions simulating the hurdles virtual patients face during clinic visits, including transportation predicaments and check-in procedures. By immersing themselves in these challenges from the virtual patient’s perspective, learners foster heightened empathy and understanding, nurturing a compassionate disposition, and mitigating implicit/explicit biases.

Hence, the seamless integration of interactive VR-based serious role-playing sessions within the mHealth app advances the learning journey for health care professionals, empowering them to elevate their clinical practices and communicate with patients in a more empathic and compassionate manner. The infusion of these engaging role-playing scenarios significantly contributes to the overarching efficacy of the mHealth app.

#### Integration of Immersive VR-Based Serious Role-Playing Modules

**Figure 6:**
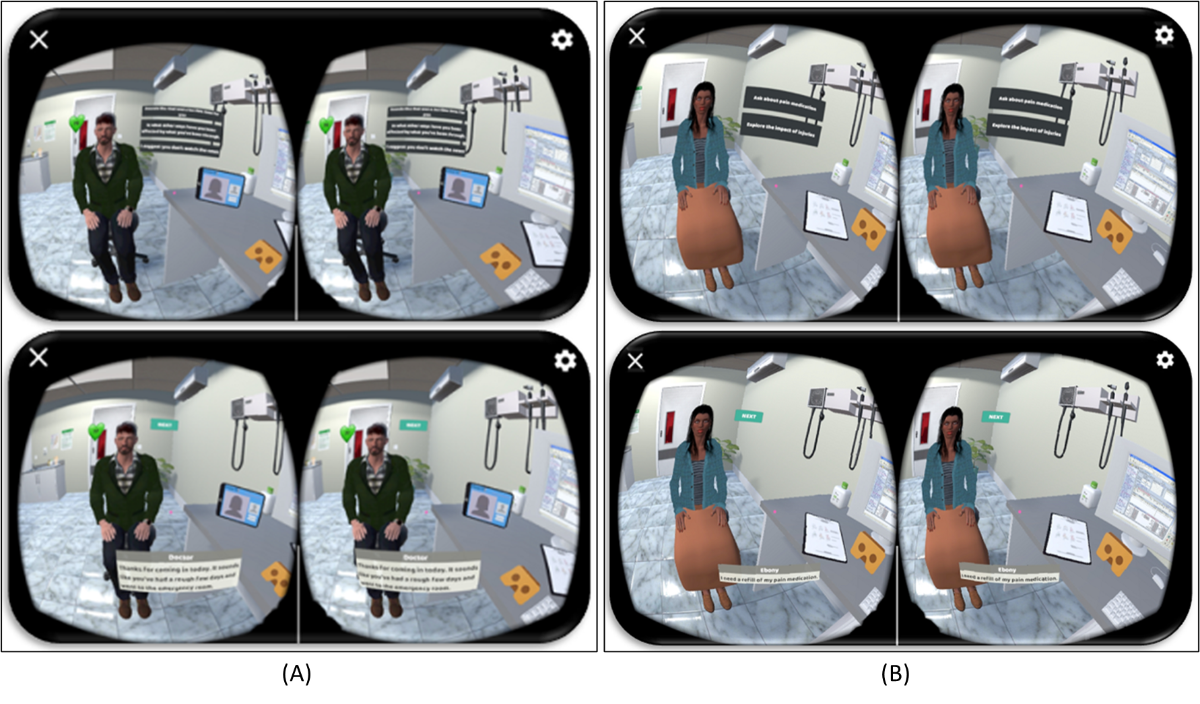
(A) Illustrations of immersive virtual reality–based serious role-playing modules of the mHealth app design in “Muhammad’s case,” and (B) Illustrations of immersive virtual reality–based serious role-playing modules of the mHealth app design in “Ebony’s case.”.

To craft a captivating and authentic clinical care practice experience for health care professionals, the immersive virtual reality–based serious role-playing modules were meticulously designed [39,40]. These modules are tailored for both Muhammad’s and Ebony’s cases, as illustrated in Figure 6A and Figure 6B, respectively. Within these modules, learners are completely immersed in a virtual environment, enabling them to interact with virtual elements and participate in clinical scenarios that provide firsthand experience in patient care practices.

By offering a first-person viewing experience from the perspective of the virtual health care provider, the module encourages active participation and enhances experiential learning. The use of 360-degree viewing capabilities in the virtual health care environment further deepens the learners’ involvement and improves their health care skills. During the immersive clinical encounter, learners are provided with a reticle pointer on the mobile device screen, enabling them to select conversational choices and interact with the virtual patient. The integration of the reticle pointer was accomplished using the Google VR software development kit, specifically the GvrReticlePointer prefab, in the app development process. However, the true power of these modules lies in their ability to evoke empathy and compassion. By immersing learners in the challenges of patient care, the learners acquire clinical skills and develop an understanding of the patient’s perspective. Thus, the immersive VR-based serious role-playing modules bridge the gap between theory and practice, offering an immersive, experiential, and empathetic approach to health care education. They equip health care professionals with the skills and perspective necessary for patient-centered care in today’s complex health care landscape.

#### Integration of Visual Cues in the mHealth App for Enhanced Learning

The assimilation of visual cues in the DEL mechanism plays a vital role in educational purposes, especially in health care–related learning tools [39,40]. In the mHealth app, visual cues serve as critical elements that help learners comprehend the various effects of their actions, interactions, and choices within the virtual case scenarios. These visual cues encompass a wide range of elements, including virtual objects, symbols, graphics, subtitles, thought bubbles, changes in virtual characters’ behavior, and facial/body emotions. By providing learners with these visual clues, the app enhances their understanding of complex scenarios, encouraging active engagement and knowledge enrichment.

A dynamic feedback system is also integrated into the serious role-playing sessions to further enhance the learning experience. This system is intricately designed to deliver immediate visual and audio responses based on the conversational choices learners make during clinical encounters with virtual patients. When selecting from response options—Apathetic, Neutral, or Empathetic—the system reacts in real time to simulate realistic patient feedback. For instance, choosing the “Empathetic” option elicits a positive response from the virtual patient, characterized by maintained eye contact, relaxed body language, and a normalized heart rate. Simultaneously, learners receive reassuring responses from the virtual patient. In contrast, opting for the “Neutral” choice results in a more reserved patient response, with the virtual patient showing slight signs of concern, which results in a moderately high range of heart rate. The dialogue becomes less enthusiastic, reflecting the impact of a neutral approach on patient interaction. Finally, selecting the “Apathetic” option triggers an extremely high increase in heart rate and a visibly distressed reaction from the virtual patient, who avoids eye contact, displays physical discomfort, and responds with unease. The dialogue, in this case, conveys the impact of apathetic communication. This dynamic feedback mechanism serves several crucial purposes. First, it offers learners a direct link between their choices and patient responses, facilitating a deeper understanding of the consequences of their communication style. Learners can witness firsthand how empathetic communication fosters a positive patient-provider relationship, while apathetic interactions can lead to patient distress. Second, the real-time nature of this feedback system provides an immersive and engaging learning experience, allowing learners to make immediate adjustments to their approach and observe the corresponding patient reactions. This iterative learning process promotes the development of essential health care skills, including empathetic communication, compassionate care, and the cultivation of unbiased attitudes toward patients. By simulating the complexities of real-world clinical encounters, this dynamic feedback system prepares health care professionals for a diverse range of patient interactions, contributing to the improvement of patient care and the promotion of patient-centric health care practices.

**Figure 7:**
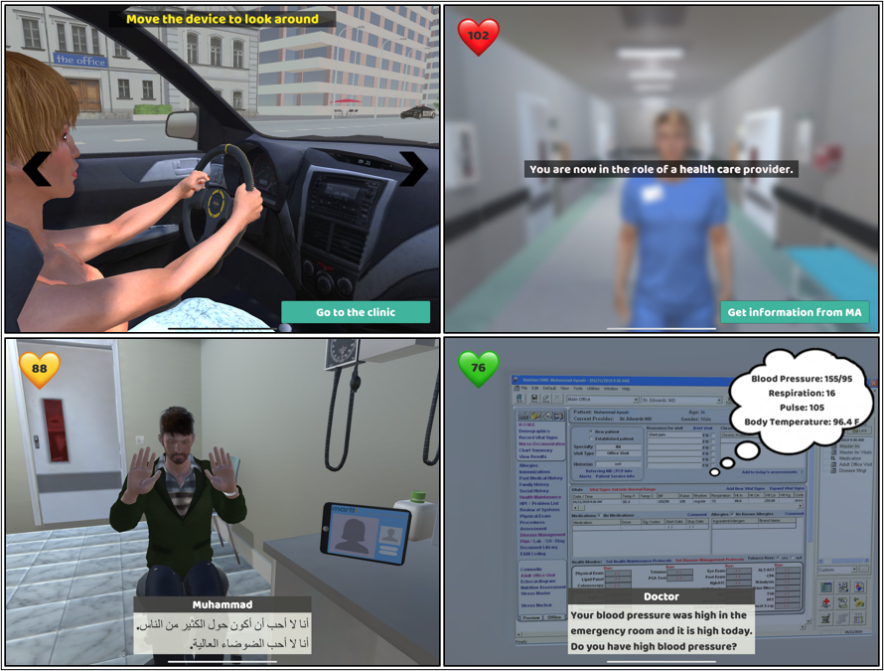
Illustration of visual cues used within the mHealth app—such as body language indicators, facial expressions, thought bubbles, dialogue boxes, and guided instructions—designed to assist users in interpreting patient emotions and making informed decisions, and reinforcing nonverbal communication skills.

Figure 7 illustrates some of the distinct visual cues, including but not limited to heart rate indicators, conversational dialogue captions, 360-degree visual capability, thought bubbles, on-screen messages, conversational audio clips, and virtual characters’ facial and body expressions, used in the app to provide learners with an immersive and effective learning experience. These visual cues serve as essential elements to enhance the assimilation of knowledge and encourage learners to develop vital skills required for health care professionals. Each of these visual cues offers specific benefits that contribute to an enriched learning process. The heart rate indicator, for instance, allows learners to gauge the emotional state of the virtual patient, aiding in understanding the impact of conversational choices. Facial and body expressions of virtual characters provide cues for emotional responses, fostering empathy and enhancing communication skills. Conversational dialogue captions enable learners to grasp the nuances of interactions, enhancing language proficiency and cultural sensitivity. The integration of 360-degree visual capability immerses learners in the virtual environment, promoting a holistic understanding of patient scenarios. Thought bubbles provide insights into the patient’s thoughts, aiding in comprehension of underlying concerns. On-screen messages guide learners through complex scenarios, facilitating step-by-step learning. Conversational audio clips engage auditory learners, reinforcing effective communication strategies. Collectively, these visual cues not only facilitate the assimilation of knowledge but also encourage learners to develop essential skills required for health care professionals, enhancing a deeper understanding of patient-care dynamics and promoting a patient-centric approach.

#### Integration of Life Course Module in the mHealth App to Explore Patients’ Life Journeys

**Figure 8:**
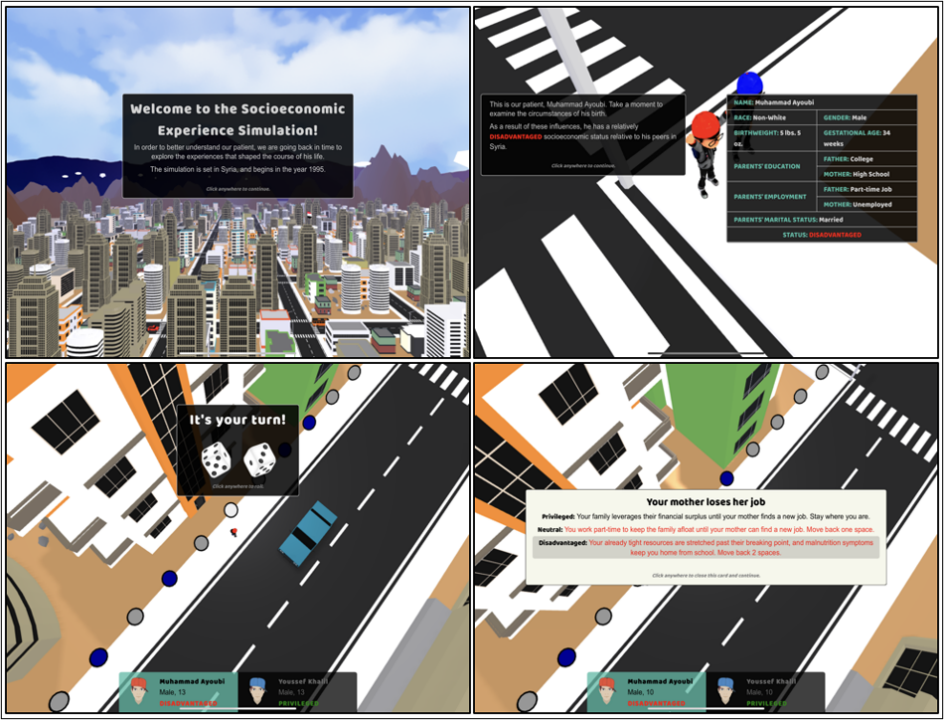
Visual representation of the integrated life course module within the mHealth app design showing virtual patient timelines and contextual influences of social determinants of health (SDH) across the lifespan.

To enrich care experiences and foster empathy and compassion toward patients, the app incorporates a life course module [39,40]. This module delves into the patient’s prior life experiences, especially those related to SDH, to provide learners with a comprehensive understanding of how these experiences impact health outcomes. By exploring both advantageous and disadvantageous life events of the patient and a privileged companion character, learners gain insights into the enduring effects of SDH on patient well-being. The life course module facilitates a connection between the learner and the patient, encouraging a deeper understanding of the individual’s unique circumstances and fostering a more empathetic approach to patient care. Figure 8 illustrates instances of the life course module in the app, providing learners with firsthand experience of the patient’s life journey and the challenges they have faced.

Incorporating the life course module adds value to the experiential learning process, equipping health care professionals with a more holistic view of patients’ lives and the impact of social determinants on their health. The module serves as a compelling educational tool, conveying valuable learning experiences to the end users. Each virtual case scenario in the mHealth app features a tailored life course module, presenting past life experiences at different age instances of the main virtual character. These events are carefully chosen to evoke emotions and supplement the learning experience. The interactive life course module features a series of illustrations that represent significant life events of the main virtual characters, creating a powerful learning environment.

By exploring the past journey of the patients through the life course module, health care professionals gain a deeper appreciation for their patients’ backgrounds, challenges, and resilience. This understanding strengthens the clinician-patient relationship, leading to improved patient care and reduced biases and health disparities. The life course module plays a vital role in instilling compassion and empathy in learners, enhancing the overall care experiences in health care settings.

Figure 8 vividly presents a series of visual illustrations drawn from the life course module, meticulously capturing pivotal events that have shaped the life journey of the main virtual character. Through these poignant depictions, learners are immersed in a poignant contrast between the life experiences of the main virtual character or patient and those of a privileged companion character. These carefully curated events, meticulously conveyed through visual cues, highlight the disparities and intricacies of various life stages, underscoring the influence of SDH on the patient’s trajectory. This unique comparison prompts learners to intimately engage with the patient’s narrative, fostering a sense of empathy, compassion, understanding, and a patient-centric perspective in their approach to health care practice. This provides a direct comparison between the main character of the simulation and another person who was more fortunate in life to provide some additional background and illustrate some of the challenges the main character is facing.

#### Easy Accessibility, Availability, Affordability, Compatibility, and Scalability

Ensuring easy accessibility, availability, affordability, and scalability of the mHealth app or serious RPG is a cornerstone of this study. Recognizing the importance of self-paced learning in enhancing educational effectiveness, the app was designed and developed to be responsive across a range of devices. The layout is optimized to accommodate various aspect ratios commonly found in iOS and Android devices, ensuring consistent usability and functionality across iPhones, iPads, Android phones, and tablets. This design prioritizes compatibility across multiple platforms, thereby maintaining a seamless user experience and enhancing accessibility and engagement for health care professionals accessing the app for skill development, anytime and anywhere with an internet connection.

An integral aspect of the app’s design philosophy is its affordability. The educational resource is intended to be accessible to all users, including health care professionals, without imposing any financial burden. Therefore, the app is made available to users at no additional cost, offering an affordable and equitable learning solution. Moreover, the scalability of the app is paramount in the overall design and implementation strategy. As the demand for health care education continues to grow, the app has been developed to be highly scalable, capable of supporting a diverse and expanding user base without compromising the quality of the learning experience.

By integrating essential elements such as accessibility, availability, affordability, and scalability, the resulting platform offers a comprehensive and user-friendly solution that empowers health care professionals to enhance their skills and knowledge effectively and efficiently. This approach also enables broader dissemination and increased reach across diverse user populations.

#### Tools and Technology

In this research, the major tools and technology used to develop the app were Unity and Reallusion Hub [42,43]. Unity, a powerful game engine, played a vital role in creating immersive VR scenarios and serious role-playing exercises. It provided real-time feedback during development, enabling lifelike simulations for enhanced learning experiences. Reallusion Hub, a comprehensive software suite for character creators, animation, and facial expression mapping, was instrumental in creating realistic avatars and enriching the simulated patient interactions within the app. The C# programming language was used to implement interactive functionalities and logic, adding dynamic elements to the app. The combination of these innovative tools and technology resulted in an innovative mHealth app or serious RPG that transformed health care professional education, offering a dynamic learning platform to empower professionals in their pursuit of improved patient care. Additionally, GitHub served as the collaborative version control platform, enabling multiple researchers and developers to work seamlessly on the project and track changes throughout the development process.

## Results

### The mHealth App’s Distribution, Data Collection, and Recruitment Method

The mHealth app is conveniently distributed through the App Store and Google Play Store in the United States, ensuring its accessibility across a wide range of iOS and Android devices, including iPhones, iPads, Android phones, and tablets. This widespread availability enables reaching a diverse user base. To thoroughly evaluate the effectiveness of the app, developed based on the DEL approach, and to gain insights into the users’ learning experiences, comprehensive pre- and postsurvey questionnaires have been integrated within the app. Table 1 shows 14 questions integrated into the assessment questionnaires of the app. Specifically, questions 1 to 9 are included in both the pre- and postsurvey or assessment questionnaires, while questions 10 to 14 are exclusive to the postsurveys or assessment questionnaires. For each question listed in Table 1, the “User Response Options or Levels” are presented as radio buttons, allowing learners to select one of the 5 distinct levels (ie, 1, 2, 3, 4, and 5) to indicate their response. These levels range from “low to high” or “never to always” or “strongly disagree to strongly agree.”

**Table 1:** Assessment or survey questions used in the analysis.

Index	Assessment or survey question	User response options or levels	Expected user response options or levels (from 1 to 5)
1	With respect to having this individual as my next patient, the amount of ANXIETY I feel is:	1 (Low) to 5 (High)	Lower is better
2	With respect to having this individual as a patient, the amount of FRUSTRATION that I feel is:	1 (Low) to 5 (High)	Lower is better
3	With respect to having this individual as a patient, the amount of COMPASSION that I feel is:	1 (Low) to 5 (High)	Higher is better
4	If given a choice, instead of this patient I would prefer to see a different patient for a routine follow-up of a chronic health problem such as hypertension.	1 (Strongly disagree) to 5 (Strongly agree)	Lower is better
5	I expect that future encounters with this patient will be:	1 (Easy) to 5 (Difficult)	Lower is better
6	I believe that this patient is largely responsible for being in their current circumstances.	1 (Strongly disagree) to 5 (Strongly agree)	Lower is better
7	I believe that the circumstances in which this patient finds themself are largely beyond their control.	1 (Strongly disagree) to 5 (Strongly agree)	Higher is better
8	I try to hide any negative thoughts about patients like this to avoid negative reactions from others.	1 (Never) to 5 (Always)	Higher is better
9	I attempt to act in nonprejudiced ways toward patients like this because it is personally important to me.	1 (Never) to 5 (Always)	Higher is better
10	I believe going through this training experience will help me decrease any negative biases I may have toward patients like this.	1 (Strongly disagree) to 5 (Strongly agree)	Higher is better
11	I believe, going through this training experience will help me be a more understanding health professional.	1 (Strongly disagree) to 5 (Strongly agree)	Higher is better
12	This activity was an effective learning platform:	1 (Strongly disagree) to 5 (Strongly agree)	Higher is better
13	I will apply at least one new thing that I learned from this simulation experience in my clinical practice.	1 (Strongly disagree) to 5 (Strongly agree)	Higher is better
14	What will you take with you into your clinical practice as a result of this simulation experience?	Enter text…(Optional)	—^a^

^a^Not applicable.

At the outset of the users’ engagement with the mHealth app, participants are guided to complete a presurvey, which functions as a baseline assessment to capture their initial understanding, expectations regarding health care interactions, and perceptions of the app’s role. This instrument serves as a calibration tool, establishing a reference point for users’ prior knowledge and attitudes. Upon completing all role-playing sessions within the app, participants are prompted to complete a postsurvey. While the presurvey establishes the baseline, the postsurvey evaluates the app’s impact by measuring knowledge acquisition, skill development, and attitudinal changes resulting from the simulated clinical experiences. This dual-survey framework enables a comprehensive evaluation of the app’s educational effectiveness. The comparative analysis of pre- and postsurvey responses provides insight into users’ progression, including improvements in health care competencies, enhanced empathetic communication, and the adoption of patient-centered, unbiased care practices.

To collect diverse learning data, primarily comprising health care professionals, a multi-faceted data collection strategy was implemented. First, in-person training sessions were conducted at local hospitals, where participants were provided with mobile devices (ie, an iPhone, iPad, Android phone, or tablet) preinstalled with the app, enabling direct engagement. This approach facilitated real-time feedback and allowed immediate resolution of any questions or concerns. Second, the app was made available through major digital platforms, specifically the App Store and Google Play Store, broadening the reach and enabling remote data collection. Additionally, a dedicated website with direct links to the app was developed, allowing users convenient access to the app page for easy download into their mobile devices.

In this study, 240 participants who voluntarily engaged with the mHealth app were evaluated based on their sustained participation throughout the sessions, demonstrating a clear willingness to contribute to the research. These participants were selected from a larger pool of volunteers who accessed the app both via web (ie, the App Store and Google Play Store) and in-person. Participants who were not evaluated could not successfully complete the entire app session, resulting in incomplete pre or postsurvey data. The unsuccessful completion of the entire session may have been due to various reasons, including technical issues like server failures, inappropriate internet connectivity, a decision to no longer participate, or simply testing the app. Hence, the analysis presented in this study corresponds to 240 participants who successfully completed the session. All descriptive and statistical calculations—including response counts, percentage derivations, CIs, effect size (Cohen *d*), odds ratios (ORs), and 1-tailed paired *t* test—were performed using Microsoft Excel 365 software (Microsoft Corporation, Redmond, Washington).

Among the 240 individuals who completed the mHealth app session, 207 (86.3%) individuals identified as female, 29 (12.1%) individuals identified as male, and 4 (1.7%) individuals identified as non-Binary. This demographic spread reflects a predominant female representation and highlights the need for further engagement strategies to support participation across gender identities.

Moreover, 36/240 (15.0%) individuals were aged between 11 and 20 years, 100/240 (41.7%) individuals fell within the 21-30 years of age, 39/240 (16.3%) individuals were aged between 31 and 40 years, 28/240 (11.7%) individuals were aged between 41 and 50 years, 25/240 (10.4%) individuals fell within the 51-60 years, 10/240 (4.2%) individuals were aged 61-70 years, and 2/240 (0.8%) individuals were in the age range of 71-80 years. This broad age spectrum highlights the inclusion of participants across generational cohorts, offering insight into how the mHealth app engaged learners at varied developmental stages and professional maturity levels.

In addition, the participant pool demonstrated a wide distribution of professional experience levels. Of the 240 individuals who completed the full app session, 95 (39.6%) individuals reported having less than one year of professional experience, followed by 47 (19.6%) individuals with 1 to 5 years, and 30 (12.5%) individuals with 6 to 10 years. A smaller subset included 20 (8.3%) individuals with 11 to 15 years of experience, while 3 equally sized groups—each with 9 (3.8%)—had between 16 and 30 years of experience, segmented into the 16- to 20-, 21- to 25-, and 26- to 30-year ranges. Finally, 21 (8.8%) individuals reported over 31 years of experience in their respective health care professions. This distribution underscores the app’s relevance across a spectrum of clinical maturity—from early-career professionals to seasoned practitioners.

Furthermore, the participant pool comprises a diverse range of health care professionals, including 18/240 (7.5%) physicians, 75/240 (31.3%) nurses, 24/240 (10.0%) social workers, 8/240 (3.3%) nurse practitioners or other types of advanced practice registered nurses, 4/240 (1.7%) physician assistants, 2/240 (0.8%) dental hygienists, and 1/240 (0.4%) each of dentist, optometrist, physical therapist, occupational therapist, medical resident, clinical psychologist, pharmacist, and respiratory therapist. Additionally, 23/240 (9.6%) were nonclinical personnel, and 78/240 (32.5%) were individuals from other relevant health care professions. This diverse composition supports a robust analysis of how the mHealth app performs across a range of professional backgrounds.

The following section provides a distinct analysis to evaluate the effectiveness of the mHealth app, with all discussions pertaining to the question indices corresponding to the questions listed in Table 1. The distinctive nature of the questions necessitated careful consideration of whether higher or lower response levels indicated improvement, tailoring the result analysis to the specific learning objectives of each question.

### Hypotheses Consideration for the Result Analysis

#### Motivation

In this research, 9 hypotheses were developed to evaluate the impact of the mHealth app on health care professionals’ skills and attitudes toward patients. Each hypothesis aligns with a specific survey question from questions 1 to 13 listed in Table 1.

#### Hypothesis 1 (Corresponding to Question 1)

The mHealth app will lead to a reduction in participants’ anxiety levels toward patients. It is hypothesized that the app’s first-person VR scenarios and role-playing exercises would offer a risk-free environment for participants to practice patient interactions, contributing to decreasing anxiety levels.

#### Hypothesis 2 (Corresponding to Question 2)

The mHealth app will lead to a reduction in participants’ frustration levels toward patients. It is hypothesized that the app’s immersive learning experiences would provide participants with effective strategies for managing frustration and stress during patient care.

#### Hypothesis 3 (Corresponding to Question 3)

The mHealth app will lead to a statistically significant improvement in participants’ compassion and empathy toward patients. It is hypothesized that the app’s first-person VR scenarios and role-playing exercises will enhance participants’ ability to understand patients’ perspectives, mirror their feelings, and demonstrate greater empathy in their interactions.

#### Hypothesis 4 (Corresponding to Questions 4 and 5)

The mHealth app will positively influence participants’ expectations of future patient encounters (corresponding to question 5) and their willingness to see the same patient again for routine follow-ups (corresponding to question 4). It is hypothesized that the app’s DEL approach will enhance participants’ confidence and preparedness for real-life patient interactions, fostering positive expectations and increasing their willingness to see the same patient for follow-up care.

#### Hypothesis 5 (Corresponding to Questions 6 and 7)

The mHealth app based on the DEL approach will positively impact participants’ beliefs about patient responsibility (corresponding to question 6) and their perceptions of the controllability of patients’ circumstances (corresponding to question 7). It is hypothesized that the app’s immersive learning experiences will raise awareness of the complexity of patient situations and promote a more empathetic and understanding approach in health care professionals.

#### Hypothesis 6 (Corresponding to Question 8)

Participants’ tendencies to hide negative thoughts about patients will be positively impacted by the mHealth app. It is hypothesized that the app’s immersive learning experiences will reinforce the importance of providing equitable and unbiased health care services, leading to reduced negative thoughts about patients.

#### Hypothesis 7 (Corresponding to Question 9)

The mHealth app based on the DEL approach will positively impact participants’ commitment to act in nonprejudiced ways toward patients. It is hypothesized that the app’s immersive learning experiences will reinforce the importance of providing equitable and unbiased health care services.

#### Hypothesis 8 (Corresponding to Questions 10 and 11)

Participants’ engagement with the mHealth app will lead to a statistically significant increase in their belief that the training experience helps decrease negative biases (corresponding to question 10) and that it helps them become more understanding health professionals (corresponding to question 11). It is hypothesized that the immersive and experiential learning provided by the app will effectively challenge and reduce negative biases that health care professionals may have toward patients, while also promoting self-awareness and empathy.

#### Hypothesis 9 (Corresponding to Questions 12 and 13)

Participants will express a positive perception of the mHealth app as an effective learning platform (corresponding to question 12) and a strong intention to apply at least one new skill or insight gained from the simulation experience in their clinical practice (corresponding to question 13). It is hypothesized that the interactive and immersive learning experiences offered by the app will be positively received by participants, enhancing their educational experience and motivating them to apply new skills and insights in their professional practice.

By formulating these hypotheses, the research aims to establish specific expectations for the analysis of participants’ responses and determine the impact of the app on achieving the intended learning outcomes and enhancing health care professionals’ skills and attitudes toward patient care.

### CI, Cohen d, and McNemar 2×2 Odds Ratios Analysis

CIs, Cohen d values, and McNemar 2×2 ORs were calculated to evaluate shifts in learner responses, using a 95% confidence level corresponding to a conventional alpha threshold of 0.05 (ie, α=0.05) for determining statistical significance. For each survey question 1-13, the change in mean score (pre vs post) with its standard deviation and 95% CI is provided. Additionally, for questions 1-9, Cohen d effect size for the standardized mean difference and a McNemar 2×2 OR (with 95% CI) based on a standard dichotomy of favorable versus nonfavorable responses are provided. As shown in Table 1, for questions 1, 2, 4, 5, and 6, favorable responses are user option levels 1-2 and nonfavorable responses are user option levels 3-5; whereas, for questions 3, 7, 8, and 9, favorable responses are user option levels 4-5 and nonfavorable responses are user option levels 1-3. Question 14 is analyzed separately due to its descriptive and optional nature.

The data indicate a measurable positive shift in learners’ compassion toward patients, alongside reduced negative perceptions and reactions. These changes suggest the app supports skill development and promotes more empathetic and constructive engagement in clinical contexts. Participants also demonstrated greater awareness of SDH and health care biases, integrating this understanding into patient-centered care scenarios presented within the simulation.

As the analysis of individual questions proceeds, incorporating the Cohen *d* values and considering the limited response levels, it is important to acknowledge that the response scale in the survey had a finite range (ie, from 1 to 5). Participants may have already selected the optimal response level within this scale in the presurvey, leaving limited room for further showcasing an improvement in the postsurvey. This indicates that participants did not experience a decline in learning during the intervention. Moreover, engagement with the app itself inherently fostered knowledge enhancement, representing a significant positive outcome. Overall, the analysis highlights how the app provides a comprehensive learning experience that supports the development of emotional intelligence and cultural competence among health care professionals.

To illustrate, for question 1 (corresponding to hypothesis 1), presurvey mean 2.40 (SD 1.12; 95% CI 2.25-2.54) decreased to postsurvey mean 2.30 (SD 1.14; 95% CI 2.16-2.44). The narrower postsurvey CI and lower mean value of participants’ responses compared with the presurvey indicate a reduced anxiety participants feel about encountering the presented patient as their next patient. The Cohen d effect size value is 0.08, reflecting the observed changes in participants’ responses. Dichotomizing participants’ responses into favorable (ie, user response levels 1-2) versus nonfavorable (ie, user response levels 3-5) yields OR 1.14 (95% CI 0.73-1.78), indicating a nonsignificant trend toward increased low-anxiety reporting.

Similarly, for question 2 (corresponding to hypothesis 2), presurvey mean 1.83 (SD 1.07; 95% CI 1.70-1.97) increased to postsurvey mean 2.08 (SD 1.14; 95% CI 1.94-2.23). The increment in the postsurvey CI and the higher mean value of participants’ responses compared with the presurvey suggest a rise in frustration when managing the presented patient. Participants may experience challenges or complexities while managing this patient, but the simulation enhances their ability to navigate such encounters. The Cohen *d* effect size value is 0.23, which demonstrates the impact of the intervention on participants’ frustration levels. Dichotomizing participants’ responses into favorable (ie, user response levels 1-2) versus nonfavorable (ie, user response levels 3-5) yields OR 0.51 (95% CI 0.31-0.83), indicating a significant decline in low-frustration reporting.

For question 3 (corresponding to hypothesis 3), presurvey mean 4.18 (SD 0.94; 95% CI 4.06-4.29) increased to postsurvey mean 4.60 (SD 0.73; 95% CI 4.51-4.69). The elevated postsurvey CI and higher mean value of participants’ responses compared with the presurvey indicate a significant improvement in learners’ compassion levels. The mHealth app effectively fosters a more empathetic response among participants, enhancing their ability to provide compassionate care to patients. The Cohen *d* effect size value is 0.50, highlighting the effect of the intervention on enhancing compassion among participants. Dichotomizing participants’ responses into favorable (ie, user response levels 4-5) versus nonfavorable (ie, user response levels 1-3) yields OR 6.17 (95% CI 2.60-14.61), indicating a strong, significant increase in compassionate responses.

Moving on to question 4 (corresponding to hypothesis 4), presurvey mean 2.15 (SD 1.19; 95% CI 2.00-2.30) decreased to postsurvey mean 1.93 (SD 1.10; 95% CI 1.79-2.07). The reduction in the postsurvey CI and reduced mean value of participants’ responses compared with the presurvey, signifies a decreased inclination among participants to see a different patient for routine follow-up, indicating greater comfort with managing such encounters. The Cohen *d* effect size value is 0.20, signifying the influence of the intervention on participants’ preferences for different patients. Dichotomizing participants’ responses into favorable (ie, user response levels 1-2) versus nonfavorable (ie, user response levels 3-5) yields OR 2.87 (95% CI 1.59-5.16), indicating a significant improvement in follow-up comfort.

For question 5 (corresponding to hypothesis 4), presurvey mean 2.87 (SD 0.98; 95% CI 2.74-2.99) decreased to postsurvey mean 2.74 (SD 1.00; 95% CI 2.61-2.87). The finer postsurvey CI and decreased mean value of participants’ responses compared with the presurvey imply greater ease in dealing with future patient encounters, indicating improved confidence in managing patient interactions. The Cohen *d* effect size value is 0.13, suggesting changes in participants’ expectations regarding future encounters. Dichotomizing participants’ responses into favorable (ie, user response levels 1-2) versus nonfavorable (ie, user response levels 3-5) yields OR 1.85 (95% CI 1.16-2.96), indicating a significant gain in confidence for managing patient interactions.

Regarding question 6 (corresponding to hypothesis 5), presurvey mean 2.02 (SD 0.98; 95% CI 1.89-2.14) decreased to postsurvey mean 1.70 (SD 0.95; 95% CI 1.58-1.82). The reduction in postsurvey CI and decreased mean value of participants’ responses compared with the presurvey suggests a change in participants’ perspectives, indicating a decreased inclination to attribute patients’ circumstances entirely to their own responsibility. The Cohen *d* effect size value is 0.33, indicating shifts in participants’ perspectives on patient responsibility attribution. Dichotomizing participants’ responses into favorable (ie, user response levels 1-2) versus nonfavorable (ie, user response levels 3-5) yields OR 2.94 (95% CI 1.70-5.10), indicating a significant shift toward reduced patient-blaming.

Conversely, for question 7 (corresponding to hypothesis 5), presurvey mean 3.51 (SD 1.02; 95% CI 3.38-3.64) increased to postsurvey mean 3.96 (SD 1.21; 95% CI 3.81-4.11). The elevated postsurvey CI and higher average value of participants’ responses compared with the presurvey signify that a greater consensus among participants that patients face circumstances that are significantly beyond their control. This alignment with the statement suggests a prevailing belief that external factors play a substantial role in shaping the patients’ situations. The Cohen *d* effect size value is 0.40, illustrating the influence of the intervention on participants’ beliefs about external control. Dichotomizing participants’ responses into favorable (ie, user response levels 4-5) versus nonfavorable (ie, user response levels 1-3) yields OR 6.00 (95% CI 3.07-11.72), indicating a robust improvement in external-factor recognition.

For question 8 (corresponding to hypothesis 6), presurvey mean 3.88 (SD 1.31; 95% CI 3.71-4.05) increased to postsurvey mean 4.03 (SD 1.31; 95% CI 3.86-4.20). The narrower postsurvey CI and higher average value of participants’ responses compared with the presurvey suggest that the app successfully encourages participants to adopt a more empathetic tactic. Participants express a stronger commitment to suppressing negative thoughts and reactions, contributing to a more positive patient-provider interaction. The Cohen *d* effect size value is 0.11, pointing to changes in participants’ efforts to suppress negative thoughts. Dichotomizing participants’ responses into favorable (ie, user response levels 4-5) versus nonfavorable (ie, user response levels 1-3) yields OR 2.22 (95% CI 1.01-4.88), indicating a significant improvement in emotional regulation.

Regarding question 9 (corresponding to hypothesis 7), presurvey mean 4.71 (SD 0.70; 95% CI 4.62-4.80) increased to postsurvey mean 4.78 (SD 0.67; 95% CI 4.69-4.86). The narrower postsurvey CI and higher average value of participants’ responses compared with the presurvey indicate that the app reinforces participants’ commitment to acting in nonprejudiced ways. Participants exhibit an increased dedication to treating patients with equity and respect, reflecting positively on their interpersonal skills. The Cohen *d* effect size value is 0.10, reflecting the impact of the intervention on participants’ commitment to nonprejudiced behavior. Dichotomizing participants’ responses into favorable (ie, user response levels 4-5) versus nonfavorable (ie, user response levels 1-3) yields OR 1.50 (95% CI 0.42-5.32), indicating a nonsignificant trend toward improved equitable attitudes.

Together, the analyses of questions 1 to 9 confirm that the mHealth app or serious game fosters a more compassionate, empathetic, and inclusive health care environment. Participants’ emotional responses and attitudes are positively influenced, contributing to improved patient-provider interactions and better patient outcomes. Additionally, responses to questions 10 to 13, which are included only in the postsurvey, further reinforce the effectiveness of the app in fostering positive changes in learners’ attitudes and behaviors.

For question 10 (corresponding to hypothesis 8), postsurvey mean 4.15 (SD 0.97; 95% CI 4.03-4.27) indicates a considerable proportion of participants agreed with the question’s statement. The mean value of the participants’ responses further emphasizes the positive impact of the simulation experience on reducing negative biases.

Similarly, for question 11 (corresponding to hypothesis 8), postsurvey mean 4.18 (SD 0.95; 95% CI 4.05-4.30) underscores a greater proportion of participants who agreed with the question’s statement. The mean value of the participants’ responses affirms the app’s role in promoting empathy and understanding among health care professionals.

Regarding question 12 (corresponding to hypothesis 9), postsurvey mean 3.85 (SD 1.14; 95% CI 3.71-3.99) indicates a positive consensus among learners regarding the app’s effectiveness as a learning platform. The mean value of the participants’ responses supports the notion that interactive simulation fosters effective learning experiences.

Last, for question 13 (corresponding to hypothesis 9), postsurvey mean 4.07 (SD 0.94; 95% CI 3.95-4.19) demonstrates learners’ commitment to applying the knowledge gained from the simulation in clinical practice. The mean value of the participants’ responses further reinforces the app’s potential to drive meaningful changes in real-world patient care.

Together, the analyses of questions 10 to 13 confirm that the app provides a comprehensive and impactful learning experience for health care professionals. It equips learners with essential skills, such as empathy, cultural humility, and self-awareness, which are vital for delivering patient-centered care. The app’s positive influence on learners’ emotional responses, attitudes, and professional development enhances the overall quality of patient care and strengthens the health care workforce’s ability to provide inclusive and equitable services.

### Analysis of Optional Descriptive Survey Question

Participants were invited to respond to an optional descriptive question (question 14) listed in Table 1 to gather qualitative insights into their experiences with the mHealth app’s simulation. Table 2 presents a selection of verbatim responses from learners or participants for question 14, highlighting their improved self-awareness, compassion, and empathy toward patients. These responses provide valuable observations that illuminate the app’s impact on their emotional connection with patients and their ability to empathize with patients’ experiences.

The learners’ feedback indicates that engaging with the simulation allowed them to develop a deeper sense of understanding and sensitivity to patients’ emotions. By imagining how patients may feel and mirroring patients’ feelings, participants demonstrated heightened emotional awareness and the capacity to connect with patients on a more acute level. This enhanced emotional connection is a crucial aspect of patient-centered care, as it fosters a stronger therapeutic alliance between health care professionals and their patients.

Furthermore, participants’ responses suggest that the mHealth app effectively cultivated a nonprejudicial attitude among learners. By putting themselves in the patient’s shoes and striving to understand the situation from the patient’s perspective, participants demonstrated a commitment to embracing diverse patient experiences and perspectives. This newfound perspective-taking ability can break down barriers and disparities in health care, promoting equitable and inclusive care practices.

The qualitative feedback provided by participants in question 14 highlights the broader impact of the app beyond quantitative metrics. While the CI analysis and mean values offer valuable insights into learners’ attitudes and self-awareness, the optional question delves into the emotional and empathetic transformations experienced by participants.

The collection of learners’ responses in question 14 validates the effectiveness of the app in fostering a more empathetic and patient-centered approach among health care professionals. It also underscores the importance of integrating emotional intelligence and cultural competence into health care education and training. By developing emotionally intelligent health care professionals, the app empowers learners to deliver compassionate, patient-centered care and contribute positively to the overall health care experience.

This qualitative analysis provides a comprehensive understanding of the diverse ways in which the app influences learners’ attitudes and emotional responses. These valuable insights complement the quantitative results, further affirming the mHealth app’s potential to transform the health care landscape positively.

**Table 2:** A few of the participants’ responses to the (an optional) survey question 14.

Index	Profession	Participant’s learning experience or comments
1	Physician	“Directly engage social worker as part of the patient centered medical home”
2	Physician	“try to remember extenuting circumstances”
3	Physician	“I will better understand the implication of adverse life experiences.”
4	Physician	“Try to understand a pt. from their perspective.”
5	Physician	“forms in languages other than English”
6	Physician assistant	“I have learned to not be biased because you never know what people are going through.”
7	Nurse	“More compassion!”
8	Nurse	“language barrier solutions”
9	Nurse	“understanding of what pt has to go through just to get to appointment”
10	Nurse	“use of simulation for my students concerning social determinants of health”
11	Nurse	“Being centered”
12	Nurse	“some patients circumstances are beyond their control”
13	Nurse	“to accomidate needs of patients. Work closely with soical workers to ensure proper followup”
14	Nurse	“Empathy”
15	Nurse	“Working to understand all aspects that go into what makes anpatient who they are”
16	Nurse	“Just how much behind thne individual can become with SDOH”
17	Nurse	“Qwait until the client broaches the topic and focus on the impact to functional life instead”
18	Nurse	“least 10 min rather then right away. let the patient calm down. we never know what someone else may be going through”
19	Nurse practitioner or other type of advanced practice registered nurse	“Look at the whole person.”
20	Dental hygienist	“Remember to respond first with empathy and ask patient before doing anything.”
21	Dental hygienist	“Not to judge anyone because you dont know their story”
22	Optometrist	“This worked well.”
23	Social worker	“I appreciate the action orientedness of the clinic. The MD saw her eventhought she was late”
24	Social worker	“Take a step back use MI and try to understand the patients unique background”
25	Social worker	“ideration”
26	Social worker	“Taking the time to ask effective questions and understand why they came in.”
27	Social worker	“Compassion and a non judgemental attitude. Always explore other options to help youe client”
28	Social worker	“People?s life circumstances are far beyond what meets the eye!”
29	Nonclinical personnel	“I will bring a better understanding and more empathy in my interactions.”
30	Nonclinical personnel	“To identify a patient’s issues rather than assuming based on previous encounters at other places.”
31	Nonclinical personnel	“Be more understanding”
32	Nonclinical personnel	“The importance of asking questions”
33	Nonclinical personnel	“compassion and understanding is extremely important”
34	Other	“I will take a renewed reminder that people dont often choose their unfortunate circumstances.”
35	Other	“putting myself in others shoes”
36	Other	“patience”
37	Other	“to be more understanding”
38	Other	“always be understanding”
39	Other	“I do not know the daily things someone is going through and always treat everyone to the best of my ability.”
40	Other	“I will not pass judgement on patients.”
41	Other	“Asking the right questions and leading with support and understanding”
42	Other	“To act in a non prejudiced way towards all patients and help everyone no matter their circumstances”
43	Other	“The overall process of each step taken place during peoples appointments.”
44	Other	“people can’t always control their circumstances”
45	Other	“Treat every patient the same without any prejudice/bias. You never know what some patients have been through.”
46	Other	“Before judging a patient i will try to put myself in their shoes”
47	Other	“I wont assume patients on opioids are drug seeking and i will try to advocate for patients”
48	Other	“I will try to understand each individual’s background to help get them the treatment they need.”
49	Other	“Be more open”
50	Other	“ls will allow as a healthcare professional to fully engage without bias and be able to further help the patient as a whole.”

### 1-Tailed Paired t Test Analysis

To further explore the effectiveness of the mHealth app, a 1-tail paired *t* test analysis was conducted on questions 1 to 9, which were present in both the pre- and postsurvey questionnaires. An alpha level of 0.05 (ie, α=0.05) was used to determine statistical significance, meaning that *P* values less than .05 were considered indicative of meaningful improvement. As depicted in Table 3, the 1-tailed paired *t* test results revealed that questions 2, 3, 4, 5, 6, 7, 8, and 9 (corresponding to hypotheses 2, 3, 4, 5, 6, and 7, respectively) demonstrated statistically significant differences between pre- and postsurvey scores, with *P* values less than .05. This suggests that the app had a substantial positive impact on learners’ responses to these questions, demonstrating improvements in various aspects, including confidence and preparedness for real-life patient interactions, positive beliefs, enhanced compassion, reduced negative thoughts, and a more nonprejudiced attitude toward patients. These findings further support the effectiveness of the app in enhancing the health care skills of learners.

**Table 3:** A 1-tail paired t test analysis between pre- and postassessment questionnaires or surveys for survey questions 1 to 9 (*P* values are rounded to nearest hundredth decimal).

Pre- and postsurvey question index	*P* value
Que-1	.11
Que-2	<.001
Que-3	<.001
Que-4	<.001
Que-5	.04
Que-6	<.001
Que-7	<.001
Que-8	.005
Que-9	.04

However, specifically for question 1 (corresponding to hypothesis 1), the 1-tailed paired *t* test results showed *P* values slightly greater than .05, indicating that the differences between pre- and postsurvey scores were not statistically significant at the 0.05 level. This outcome is likely influenced by the fact that the participants already gave near-optimal scores for question 1 in the presurvey, leaving limited room for measurable improvement. Nonetheless, the mHealth app showed positive trends in this aspect, but due to the limited room for improvement, the result (for question 1 or hypothesis 1) was not strong enough to reach statistical significance in a tailed paired *t* test analysis.

Overall, the combination of CI analysis and the 1-tail paired *t* test provides valuable insights into the impact of the mHealth app on learners’ experiences and skills. It demonstrates the app’s effectiveness in enhancing learners’ compassion, empathy, and nonprejudiced attitudes toward patients. The results suggest that the mHealth app can be a valuable tool in improving health care professionals’ performance and providing better care experiences and outcomes for patients.

### Additional Statistical Analysis

This section presents a comprehensive analysis of participants’ responses to the survey questionnaires associated with the app, which is designed to enhance health care professionals’ skills and promote health equity. The analysis of pre- and postsurvey data offers valuable insights into participants’ learning experiences and the app’s overall efficacy. Notably, as depicted in Table 4, for each survey question 1 to 9, 3 types of potential outcomes or responses were considered: (1) “learning improvement” or “positive response” which suggests the participant’s improved or positive learning experience examined in the postsurvey response compared with the presurvey response, (2) “neutral response” which suggests the participant’s neutral or no change learning experience examined in the postsurvey response compared with the presurvey response, and (3) “learning deterioration” or “negative response” which suggests the participant’s deteriorating or negative learning experience examined in the postsurvey response compared with the presurvey response.

**Table 4:** Response counts based on the response type for survey questions 1 to 9.

Response type	Participants’ response count								
	Que 1	Que 2	Que 3	Que 4	Que 5	Que 6	Que 7	Que 8	Que 9
Positive response or learning improvement	84	50	86	72	77	90	106	49	21
Neutral response	101	113	141	136	103	122	100	161	210
Negative response or learning deterioration	55	77	13	32	60	28	34	30	9

Exploring statistical findings and their implications provides insights into the app’s capacity to cultivate compassion, empathy, and nonprejudiced attitudes toward patients. Furthermore, the app’s role in enhancing health care skills and fostering a deeper understanding of patient perspectives is also examined. This comprehensive assessment illustrates the multifaceted nature of participants’ learning behaviors, offering valuable guidance for health care education and practice.

Participants’ advancement in learning was examined by categorizing responses into “positive” (indicating improvement) and “negative” (indicating deterioration) outcomes for survey questions 1 to 9. Neutral responses were intentionally excluded to calculate improvement rates based solely on participants who exhibited a change in their response level between the pre- and postsurvey on the 5-point scale (ie, user response levels or options). This approach focuses on visible shifts in attitudes and perceptions, while recognizing that it does not capture potential learning reinforcement among participants whose responses remained unchanged.

Notably, for question 3 (corresponding to hypothesis 3), 86/99 (87%) participants—excluding 141 neutral responses—demonstrated enhanced compassion and empathy toward patients. This underscores the mHealth app’s success in nurturing emotional intelligence and effective care competencies among health care professionals.

Additionally, questions 6 and 7 (corresponding to hypothesis 5) revealed substantial growth, focusing on how participants perceive patient circumstances. For question 6, 90/118 (76.3%) participants demonstrated positive change, characterized by a reduced tendency to attribute blame to the patient for their situation. For question 7, 106/140 (75.7%) participants showed improvement, indicating heightened recognition that patients may be influenced by external, uncontrollable factors. These shifts reflect strengthened bias awareness and a deeper understanding of social determinants in clinical encounters, highlighting the mHealth app’s effectiveness in enhancing these skills and attitudes.

Similarly, questions 1, 4, 5, 8, and 9 (corresponding to hypotheses 1, 4, 6, and 7) exhibited substantial learning improvement. Specifically, for question 1, 84/139 (60.4%) participants reported reduced anxiety toward patients. Question 4 showed that 72/104 (69.2%) participants felt more comfortable engaging the same patient for follow-up care. Question 5 reflected increased confidence in managing patient interactions, with 77/137 (56.2%) participants demonstrating positive change. For question 8, 49/79 (62%) participants reported stronger efforts to suppress negative thoughts, while question 9 revealed that 21/30 (70%) participants expressed a heightened commitment to providing nonprejudiced care. Collectively, these findings highlight the mHealth app’s effectiveness in strengthening core aspects of health care professionalism, including emotional regulation, bias mitigation, and patient-centered attitudes.

Conversely, question 2 (corresponding to hypothesis 2) demonstrated a comparatively lower rate of learning improvement, with 50/127 (39.4%) participants showing positive change in their frustration levels toward patients, after excluding 113 participants who provided neutral responses. While the improvement percentage was modest relative to other outcomes, the simulation still delivered meaningful attitudinal shifts in how participants engaged with emotionally challenging scenarios—underscoring its value in navigating emotionally charged patient interactions.

This analysis provides a holistic view of participants’ learning improvement across various facets of the health care profession. The significant positive changes observed in key areas underscore the effectiveness of the mHealth app in fostering compassion, empathy, and nonprejudiced attitudes among health care professionals, contributing to improved patient care and health care outcomes.

Furthermore, an additional analysis was conducted to more comprehensively evaluate participants’ learning improvement by combining positive (learning improvement) and neutral (or no change) responses versus negative (learning deterioration) responses across survey questions 1 to 9. For this analysis, neutral responses were grouped with positive ones to reflect any beneficial impact—even if a participant’s response remained unchanged between the pre- and postsurvey. This approach provides a broader understanding of how the app contributed to learning, capturing both growth and knowledge reinforcement.

Neutral responses represent an important dimension of learning enhancement and should not be overlooked when interpreting participants’ outcomes. In many cases, neutral responses occurred because participants selected optimal ratings—such as “Strongly agree” or “Strongly disagree” respectively to the question—in both the presurvey and postsurvey phases. This pattern suggests they entered the simulation with a solid foundation of knowledge or attitudes relevant to the measured construct. While these participants may not have shown numerical change, the mHealth app likely played a valuable role in reinforcing their understanding, leading to a consolidation of competencies that are essential for clinical practice.

Moreover, the design of the response scale, ranging from 1 to 5, inherently limits how participants can express marginal or qualitative gains. For individuals who already selected the highest possible rating in the presurvey, the postsurvey offered no room for upward movement—even if they did experience deeper insight, stronger commitment, or emotional growth. This structural constraint is important to consider, as it suggests that reported improvement rates may underrepresent the true impact of the simulation on highly engaged or experienced learners. As such, the neutral category includes individuals whose learning trajectory remained positive, even if not numerically reflected in postsurvey scores. Taken together, the following analysis underscores the app’s positive impact on participants’ learning experience, capturing both measurable improvement and reinforcement of existing knowledge.

Analyzing the results reveals that the mHealth app had a strong positive impact on participants’ learning experience across all survey items. Notably, for question 3 and question 9—corresponding to hypotheses 3 and 7, respectively—227/240 (94.6%) participants and 231/240 (96.3%) participants demonstrated learning improvement, demonstrating the high effectiveness of the mHealth app in fostering compassion, empathy, and promoting nonprejudiced behavior among health care professionals toward patients.

Similarly, questions 4, 6, and 8—corresponding to hypotheses 4, 5, and 6—yielded strong outcomes, with participants showing marked learning enhancement. For question 4, 208/240 (86.7%) participants showed improvement by expressing reduced preference to avoid follow-up care with the same patient. Question 6 demonstrated positive change in 212/240 (88.3%) participants, indicating diminished tendencies to blame the patient for their circumstances and increased recognition of external influences. For question 8, 210/240 (87.5%) participants shifted away from masking negative thoughts, suggesting greater internal emotional regulation and growing comfort with expressing compassionate care authentically. These findings underscore the mHealth app’s ability to support reflective attitude shifts across emotionally and ethically complex dimensions of clinical practice.

Questions 1, 2, 5, and 7—corresponding to hypotheses 1, 2, 4, and 5—also demonstrated substantial learning improvement. Specifically, for question 1 (reduced anxiety toward patients), 185/240 (77.1%) participants showed improvement. Question 2 (reduced frustration) saw gains in 163/240 (67.9%) participants, while question 5 (confidence in future patient encounters) showed progress in 180/240 (75.0%) participants. For question 7, 206/240 (85.8%) participants demonstrated increased recognition of external factors influencing patients’ circumstances. Together, these findings reflect meaningful shifts in participants’ ability to manage difficult emotions, anticipate future patient interactions more positively, and appreciate the broader social context of patient care—areas critical to effective, empathetic clinical practice.

Thus, the findings reflect the positive impact of the app on participants’ learning experience, encompassing both learning improvement and consolidation of existing knowledge. Analyzing the users’ responses provides valuable insights into the overall effectiveness of the app in advancing health care professionals’ skills and attitudes, even for participants who may have already possessed some knowledge on the subject matter.

Additionally, Table 5 offers a comprehensive view of participants’ overall response distributions across all levels (1 to 5) for both presurvey and postsurvey questions 1 through 9. This tabulation reinforces the effectiveness of the mHealth app by illustrating broader shifts in user sentiment and competence. It is important to clarify that Tables 4 and 5 represent distinct analytical approaches. Table 4 evaluates individual participants’ directional changes, categorizing their responses as “positive (improvement), neutral (no change), or negative (deterioration)” based on pre- and postsurvey comparison. In contrast, Table 5 aggregates total counts obtained for each user response option or level (ie, 1 to 5), offering a macro-level snapshot of the entire cohort’s scoring trends without pairing responses per participant. Together, these tables enrich the overall interpretation of learning impact, combining both individual-level transformations and collective score movements.

**Table 5:** Participants’ response counts for each user response level or option for pre- and postsurvey questions 1 to 9.

User response option or level	Participants’ response counts																	
	Pre-Q1	Post-Q1	Pre-Q2	Post-Q2	Pre-Q3	Post-Q3	Pre-Q4	Post-Q4	Pre-Q5	Post-Q5	Pre-Q6	Post-Q6	Pre-Q7	Post-Q7	Pre-Q8	Post-Q8	Pre-Q9	Post-Q9
1	62	77	125	99	4	4	98	113	25	27	90	132	10	14	20	21	4	4
2	69	59	57	60	11	0	50	63	49	70	76	67	20	19	25	18	0	0
3	72	69	40	54	32	12	62	40	108	90	58	29	93	40	28	23	11	9
4	26	25	9	16	85	56	17	16	49	44	12	6	72	57	58	49	31	19
5	11	10	9	11	108	168	13	8	9	9	4	6	45	110	109	129	194	208

## Discussion

### Principal Findings

This study evaluated a novel mHealth app built on a DEL approach aimed at enhancing health care professionals’ competencies and fostering health equity. Among 240 participants, the app demonstrated statistically significant improvements in multiple skill domains—including compassion, empathy, inclusive communication, confidence in patient interactions, nonprejudiced attitudes, bias awareness, and understanding patients’ perspectives. Evaluation of pre- and postsurvey responses using CI analysis, Cohen *d* effect sizes, ORs, 1-tailed paired *t* tests (α=0.05), and descriptive categorizations reinforcing the positive impact of the intervention.

The DEL approach’s strength lies in immersing learners in interactive hypothetical virtual case scenarios using a first-person perspective. Participants experienced emotionally nuanced simulations designed to challenge their clinical assumptions and encourage reflective learning. The findings underscore the effectiveness of this approach in shifting learners’ perspectives on SDH, patient responsibility attribution, and the relevance of cultural humility—all foundational to delivering equitable, patient-centered care. Moreover, the results demonstrate the significance of addressing biases and constraints in health care skills to foster health equity and improve health outcomes.

Moreover, the app’s fully automated and self-paced nature allowed participants to engage without instructor supervision, increasing accessibility and flexibility for professional development. The results reflect substantial gains in cognitive understanding and effective learning domains—validating the app’s potential as a scalable tool for health care education.

### Comparison to Prior Work

Building upon initial pilot efforts [39,40], this study demonstrates significant advancements in both scale and analytical rigor. The prior version of the DEL-based mHealth app introduced foundational concepts through role-playing scenarios and a life course module; however, its scope was limited in terms of sample size, depth of evaluation, and data transparency. This study marks a prominent evolution, involving a robust sample of 240 health care professionals and using a comprehensive suite of statistical analyses of participants’ responses for the pre- and postsurvey questionnaires, including 1-tailed paired *t* tests, ORs, CI estimations, and Cohen *d* effect sizes to rigorously assess the app’s effectiveness and impact in the field.

In terms of educational design, this iteration features enhanced backend workflow integration, improved user interface reliability, and refined survey instruments aligned with clearly defined hypotheses. These upgrades enabled more accurate and meaningful tracking of learner outcomes, particularly in empathy, cultural humility, bias awareness, and clinical preparedness. Additionally, this study also offers a deeper exploration of app features and their role in shifting provider attitudes toward patient-centered care and equity.

Collectively, the expanded scale, methodological sophistication, and validated learning outcomes underscore the strong potential of this updated mHealth app to transform health care education. It moves beyond proof of concept and into evidence-based innovation, positioning this study as a scalable, self-directed resource capable of addressing systemic challenges in clinical training and professional development.

### Strengths and Limitations

One of the primary strengths of this study lies in its integration of DEL approach into an mHealth platform, allowing health care professionals to engage with emotionally nuanced, interactive simulations in a self-directed, unsupervised manner. By offering a fully automated experience with realistic virtual case scenarios and first-person perspectives, the app fosters compassion, cultural humility, bias awareness, and reflective decision-making—all foundational to patient-centered care. Its accessibility across varying schedules and environments encourages continuous professional development, while the use of multiple analytical methods—including 1-tailed paired *t* tests, CI estimation, and effect size calculations—enhances the credibility and rigor of the evaluation. The statistically robust analysis of 240 participants adds further credibility to the intervention’s effectiveness across diverse skill domains. The app’s design—centered around virtual case-based simulations—created meaningful opportunities for learners to reflect on their beliefs, assumptions, and behaviors in patient care. The app’s ability to deliver effective and cognitive learning at scale positions as a powerful, innovative tool for advancing health equity and transforming clinical education.

This study encountered a few limitations that could be addressed in future research. The mHealth app requires an internet connection to complete the simulation, which may limit accessibility for individuals with unreliable internet connectivity. This requirement necessitates further investigation into alternative delivery methods or offline capabilities to ensure broader access to the educational content. Additionally, the current version offers only 2 hypothetical case scenarios, which may not adequately capture the full spectrum of real-world health care challenges. Incorporating a wider range of cases with varied cultural, ethical, and clinical contexts would improve realism and learner engagement. Furthermore, the conversational context choices within the app are hardcoded, lacking the flexibility and adaptability of artificial intelligence and natural language processing technologies. Incorporating these technologies could significantly enhance the app’s ability to respond dynamically to user inputs, offering a more personalized and engaging learning experience. These limitations underscore the need for future research and development efforts to address barriers to accessibility, diversify and enhance the realism of learning scenarios, and leverage advanced technologies to boost the app’s effectiveness and user satisfaction.

### Future Directions

Building on these findings, future work will focus on expanding the library of scenarios to represent a broader range of cultural contexts, clinical conditions, and ethical dilemmas. Optimizing system performance to reduce memory use and support offline access will increase usability across varied practice settings. Incorporating conversational intelligence via natural language processing and adaptive feedback mechanisms could transform the experience into a truly personalized learning environment.

Beyond technical upgrades, future DEL-based apps might integrate real-time expert guidance, augmented reality overlays, or gamified challenges to reinforce engagement and longitudinal learning. Similar virtual storylines or serious RPGs could be adapted for nursing, public health, or interprofessional education.

Ultimately, this research lays the groundwork for reimagining health care training through mobile experiential learning. By embedding equity-focused competencies into scalable platforms, such tools can contribute meaningfully to transforming how providers learn, reflect, and care—bringing us closer to a more empathetic, inclusive, and effective health care system.

## Abbreviations

DEL: digital experiential learning
mHealth: mobile health
NHE: National Health Expenditure
OR: odds ratio
RPG: role-playing game
SDH: social determinants of health
VR: virtual reality
